# Supplementary material for: The Effectiveness of Free Face Mask Distribution on Use of Face Masks. A Cluster Randomised Trial in Stovner District of Oslo, Norway
Source: Int J Environ Res Public Health. 2021 Aug 26;18(17):8971. doi: 10.3390/ijerph18178971 (PMC8430712; doi:10.3390/ijerph18178971)
Supplement: Supplementary file 1 [file ijerph-18-08971-s001.zip › File S1 Study protocol.pdf]

Version 30.04.2021

The Effectiveness of Distributing Free Face Masks to Increase Use of Face Masks. A Cluster Randomised Experiment in Stovner District, Oslo, Norway (Study Protocol)

Atle Fretheim, Centre for Informed Health Choices, Norwegian Institute of Public Health

Ingeborg Hess Elgersma, Health Services Division, Norwegian Institute of Public Health

Fredrik Aaeng Kristiansen, Research Administrative Support, Norwegian Institute of Public Health

Caroline Rømming Varmbo, Stovner District-administration, City of Oslo, Norway

Miriam Kristine Salame Olsbø, Stovner District-administration, City of Oslo, Norway

Martin Flatø, Centre for Fertility and Health, Norwegian Institute of Public Health

Corresponding author: atle.fretheim@fhi.no

ABSTRACT

Design: We will carry out a cluster randomised trial of free face mask distribution in Stovner District, Oslo, Norway. Grocery stores will serve as study sites.

Methods: Over three weeks the stores will be randomised daily to having people outside the entrance, who hand out face masks to customers. We will compare the proportion of customers who wear face masks as they enter the stores, with or without face mask distribution.

INTRODUCTION

Background

In most countries, face masks are recommended as a means of reducing the spread of COVID-19, especially in settings where the risk of transmission is perceived as high. While many constituencies have mandated the use of face masks, others have issued recommendations and relied on the public's volunteer use of masks.[1]

In Norway there has been no tradition for using face masks in the community and in the earliest phase of the COVID-19 pandemic face masks were not recommended by the health authorities. However, this has gradually changed, and since October 2020 Oslo has made use of face masks mandatory in stores, on public transport etc. where social distancing is not feasible. Children (under 12 years) are excluded from the mandate.

A year into the COVID-19 pandemic, few if any studies have been conducted to assess the effectiveness of face mask-promoting interventions. A few studies have explored how various forms of messaging or whether making their use mandatory, influences people's intentions to wear face masks,[2 3] but there have been no intervention studies where actual use of face masks has been monitored – as far as we know.

In other words, science has little to offer public health officers and others tasked with effectively implementing face mask-use in the community.

One common-sensical approach to encouraging the use of face masks is free distribution. This strategy has some scientific backing from studies of making other types of commodities available for free or at a low price, e.g. condoms for youth.[4] However, it is far from obvious that the effectiveness of free mask distribution is likely to be similar to the effectiveness of distributing free condoms.

Stovner District in Oslo, Norway, is one of the areas of Norway that has been hardest struck by the pandemic, with around 10% of the population having tested positive for SARS-CoV-2 by the end of April 2021.[5] The local authorities have implemented several measures for infection control, including free mask distribution by engaging youth who serve as "corona hosts" at key locations. One of the main tasks of the corona hosts is to hand out face masks. Since it is uncertain whether this form of face mask distribution has an impact on face mask use, the Norwegian Institute of Public Health teamed up with the Stovner District-administration to carry out a randomised trial of their free distribution scheme.

Objective

The aim of this study is to assess to what extent free distribution of face masks outside grocery stores leads to increased use of face masks among customers.

METHODS

Trial registration

ClinicalTrials.gov Identifier: NCT04866589

Setting

Grocery stores in Stovner District will serve as study sites. We expect to include 8-10 stores that have had no, or only sporadic, distribution of face masks previously. The trial will be conducted between 1600h and 1800h, on weekdays over three weeks, starting May 3rd, 2021. For each day of the trial the

stores will be randomised to intervention or control (i.e. no free distribution of face masks). There are two national holidays during this period, so the total numbers of study days will be 13.

Intervention

Local citizens are recruited by the district administration to serve as corona hosts and hand out face masks for free outside the entrances of stores.

Randomisation procedure

The team members from the Stovner district-administration will send a numbered list to Norwegian Institute of Public Health-team, with the names of the stores. The ordering of the stores will define the numbers used to represent each store in the randomisation process. Using Stata software, we will carry out 13 separate and independent randomisations, each assigning the intervention to half the included stores. An additional randomisation to the treatment or control group will be carried out for a marginal store each day in the event of an odd number of stores.

Unless it proves too logistically cumbersome, we will also randomise the corona hosts, i.e. the store they are allocated to will be decided through randomisation.

Analysis

We will use a linear probability model with individual use of facemask as outcome, and control for grocery store and day. Standard errors will be clustered at the grocery store level.

Outcome measurement

Our main outcomes measure will be the proportion of people wearing a mask (without distinguishing between correct or incorrect use).

As a secondary outcome we will assess the proportion of people wearing a face mask correctly (i.e. covering both mouth and nose).

We will have observers placed inside the entrance of the stores for the full duration of the trial, i.e. every weekday from 1600h to 1800h. In stores with face mask distribution, the observation point will be after passing the distribution point. They will serve as data-collectors, by counting the number of individuals entering the store, and note whether they a) wear a mask correctly, b) do not wear a mask, or c) wear a mask incorrectly. We will use a pragmatic definition of correct face mask use, i.e. that the mask covers mouth and nose.

The observers, hosts, and grocery staff will be wearing facemasks at all locations during the trial.

Only youth and adults (12 years or older) will be counted. The assessment of whether a person is a child (younger than 12 years old) and therefore not to be counted, will be based on the observer's visual judgement.

We will also collect data on the number of face masks handed out during the study period.

Sample size estimation

Based on informal reports from the Stovner District-administration, we assume that around 80% of those entering the stores will be using a face mask without free mask distribution, and a standard deviation of +/- 10% in means is assumed. If free distribution increases the use of face masks to 90%, we estimate that a trial over the planned study period will be enough to, with reasonable certainty, demonstrate a real difference (5% significance level, 80% power). The estimate is based on simulation exercises, taking into account that each randomised draw is out of the same pool of

stores, and that half the variation in means is within stores. During the trial, we will assess whether our assumptions seem to hold. If they do not, we will consider adjusting the duration of the trial.

Ethics and privacy issues

This project does not fall under the Norwegian Health Research legislation as it is not health research as per the definition in the law. Consequently, there is no need for formal ethical approval. All data we will collect is anonymous, so no data protection measures are necessary.

This research follows ethical guidelines on research in social sciences, established by the Norwegian research ethics committee.[6] Accordingly, when personal data is not collected, the requirement for informed consent of participants can be levied when the research does not imply direct contact with the participants, where the data being processed is not particularly sensitive, and where the utility value of the research clearly exceeds any disadvantages for the individuals involved. This research fulfils those criteria and hence no consent will be collected.

Participants will be informed about the study through posters at the store exit. Observers will point participants to relevant information material if approached. The information material will provide information about the purpose of the research, who has funded the project, the nature of the collected information, and dissemination plans for the results, It will also provide contact details to project management.

Findings from the study will be disseminated through mass media outlets. Researchers will make themselves available for engagement with Stovner District-administration and the community to disseminate results and discuss implications of the research.

REFERENCES

1. Felter CB, N. Which Countries Are Requiring Face Masks? : Council on Foreign Relations; 2020 [updated 04.08.2020. Available from: https://www.cfr.org/in-brief/which-countries-are-requiring-face-masks accessed 16.04. 2021.
2. Capraro V, Barcelo H. Telling people to "rely on their reasoning" increases intentions to wear a face covering to slow down COVID-19 transmission. *Appl Cogn Psychol* 2021 doi: 10.1002/acp.3793 [published Online First: 2021/04/07]
3. Betsch C, Korn L, Sprengholz P, et al. Social and behavioral consequences of mask policies during the COVID-19 pandemic. *Proc Natl Acad Sci U S A* 2020;117(36):21851-53. doi: 10.1073/pnas.2011674117 [published Online First: 2020/08/21]
4. Wang T, Lurie M, Govindasamy D, et al. The Effects of School-Based Condom Availability Programs (CAPs) on Condom Acquisition, Use and Sexual Behavior: A Systematic Review. *AIDS Behav* 2018;22(1):308-20. doi: 10.1007/s10461-017-1787-5 [published Online First: 2017/06/19]
5. Statusrapport Covid-19 Tirsdag 27. april 2021. Oslo, Norway: Oslo kommune, 2021.
6. Hvinden B, Bang K, Fjørtoft K, et al. Guidelines for Research Ethics in the Social Sciences, Humanities, Law and Theology. Oslo, Norway: The National Committee for Research Ethics in the Social Sciences and the Humanities (NESH) 2019.
