# Supplementary material for: The Effectiveness of Free Face Mask Distribution on Use of Face Masks. A Cluster Randomised Trial in Stovner District of Oslo, Norway
Source: Int J Environ Res Public Health. 2021 Aug 26;18(17):8971. doi: 10.3390/ijerph18178971 (PMC8430712; doi:10.3390/ijerph18178971)
Supplement: Supplementary file 1 [file ijerph-18-08971-s001.zip › Table S1.pdf]

Table S1. Descriptive statistics by store, weighted by number of observations.

	Face mask distribution	Number of days	Wearing mask (%)	Wearing mask correctly (%)	Customers per day (mean)
Store 1	Distribution	5	98.6	98.5	208
	No distribution	8	93.6	92.5	240
Store 2	Distribution	11	95.4	94.0	108
	No distribution	2	91.5	89.1	101
Store 3	Distribution	6	98.2	97.2	281
	No distribution	7	95.3	93.1	320
Store 4	Distribution	7	97.0	92.5	254
	No distribution	6	87.8	81.7	297
Store 5	Distribution	8	97.7	95.9	148
	No distribution	5	90.6	88.2	131
Store 6	Distribution	5	96.2	94.4	65
	No distribution	8	91.5	90.8	72
Store 7	Distribution	5	92.8	90.5	53
	No distribution	8	72.7	68.2	59
Store 8	Distribution	5	96.0	92.3	367
	No distribution	8	93.0	90.5	400
Store 9	Distribution	9	98.8	97.4	145
	No distribution	4	93.4	90.5	142
Total	Distribution	61	97.2	95.0	221
	No distribution	56	91.7	88.4	281
