# Supplementary material for: The Effectiveness of Free Face Mask Distribution on Use of Face Masks. A Cluster Randomised Trial in Stovner District of Oslo, Norway
Source: Int J Environ Res Public Health. 2021 Aug 26;18(17):8971. doi: 10.3390/ijerph18178971 (PMC8430712; doi:10.3390/ijerph18178971)
Supplement: Supplementary file 1 [file ijerph-18-08971-s001.zip › Table S2.pdf]

Table S2. Results, main regression and sensitivity checks.

**Sample →**	**Full sample**				**Days 4, 5, 7, 8, & 10-13**	
Dependent variable →	Mask usage	Correct usage	Mask usage	Correct usage	Mask usage	Correct usage
	(1)	(2)	(3)	(4)	(5)	(6)
Face mask distribution	6.00***	7.25***	5.84***	7.06***	6.03***	6.78***
	(1.07)	(1.14)	(0.99)	(1.04)	(0.86)	(0.93)
Observations per cluster			-0.01	-0.01		
			(0.01)	(0.01)		
Store controls	Yes	Yes	Yes	Yes	Yes	Yes
Day controls	Yes	Yes	Yes	Yes	Yes	Yes
R2	0.6749	0.6893	0.6812	0.6945	0.7288	0.7552
N (clusters)	117	117	117	117	72	72
n (individuals)	21,524	21,524	21,524	21,524	12,942	12,942

All numbers are per 100. Standard errors clustered at the store level in parenthesis. * p<10%, ** p<5%, ***p<1%
