# Supplementary figures and images for: The Effectiveness of Free Face Mask Distribution on Use of Face Masks. A Cluster Randomised Trial in Stovner District of Oslo, Norway
Source: Int J Environ Res Public Health. 2021 Aug 26;18(17):8971. doi: 10.3390/ijerph18178971 (PMC8430712; doi:10.3390/ijerph18178971)

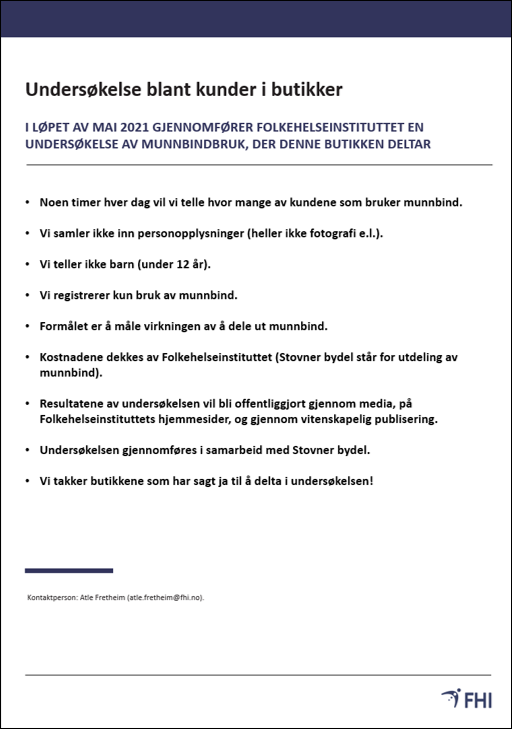

Supplement: Supplementary file 1 [file ijerph-18-08971-s001.zip › File S3 Information poster (in Norwegian).png]
